# Factors Affecting Hair Cortisol Concentration in Domestic Dogs: A Focus on Factors Related to Dogs and Their Guardians

**DOI:** 10.3390/ani15131901

**Published:** 2025-06-27

**Authors:** Chiara Mariti, Giulia Russo, Chiara Mazzoni, Carmen Borrelli, Eleonora Gori, Verena Habermaass, Veronica Marchetti

**Affiliations:** Department of Veterinary Sciences, University of Pisa, 56124 Pisa, Italy; giulia.russo@phd.unipi.it (G.R.); kymazz83@yahoo.it (C.M.); carmen.borrelli@phd.unipi.it (C.B.); eleonora.gori@unipi.it (E.G.); verena.habermaass@phd.unipi.it (V.H.); veronica.marchetti@unipi.it (V.M.)

**Keywords:** hair, cortisol, dog, dog guardian, welfare, stress

## Abstract

Considering the controversial findings in the existing literature, this study aimed to deepen the knowledge about Hair Cortisol Concentration (HCC) in dogs by evaluating the influence on HCC of some factors related to dogs and guardians. Hair was collected from two groups: 128 Healthy Dogs (HD) and 25 dogs with a primary Chronic Gastroenteric Disease (CGD). Guardians of HD filled in a questionnaire, including dogs and respondents’ demographic information, and the perceived welfare and behaviour of the dog. HCC were measured with an enzyme immunoassay kit. A Wilcoxon rank-sum test was conducted to compare HCC in HD and CGD. For HD, a multiple linear regression and an ordinary logistic regression were performed with the dependent variable being HCC and the independent variables being dog and guardian characteristics and the questionnaire evaluations. HCC was statistically lower in CGD (medians: 4.79 versus 6.41 pg cortisol/mg hair; W = 961, *p* < 0.001). A positive association between HCC and guardian’s age was found (β: 0.012; t-value = 3.205; *p* < 0.01). Previous literature has shown that several factors can affect HCC in dogs; however, given the controversial results, a large sample and a multiparametric analysis, as in this study, can advance knowledge and highlight newly investigated factors. This study revealed the importance of also considering factors related to the guardian and the possibility that multiple factors interact and collectively influence HCC.

## Simple Summary

Identifying all the factors that might influence hair cortisol concentration in dogs remains challenging. In this study, we aimed to determine which characteristics may affect cortisol levels in dog hair, as this is a commonly used method for assessing cortisol concentration over an extended period as a possible indicator of chronic stress. We found that dogs affected by a gastroenteric chronic disease had lower cortisol levels in their hair compared to healthy dogs, and that cortisol levels in dogs’ hair increased with the increase of dogs’ age as well as guardians’ age. This highlights the importance of considering both the dog’s and the guardian’s characteristics in future studies, as these could impact dog welfare and/or may interact with stress levels to determine cortisol concentrations.

## 1. Introduction

The role of cortisol as an acute stress indicator is well-established; however, in veterinary medicine, ongoing efforts aim at identifying reliable methods for measuring cortisol and correlating it with chronic stress levels, in order to expand the understanding of the internal and external factors that influence it [1]. Generally, cortisol measurements are based on the analysis of blood, saliva, urine, and faeces, which reflect short-term (e.g., blood, saliva, urine) or medium-term (faeces) variations; however, these present several limitations. For instance, they are susceptible to the effects of sampling procedure stress and of circadian rhythms (e.g., blood, saliva) [2,3,4,5]. Indeed, these “short-term” matrices can be affected by considerable daily physiological fluctuations, which complicates the accurate assessment of long-term systemic cortisol levels [2,6]. Moreover, salivary cortisol presents some limitations that may compromise its reliability as an indicator of animal welfare; for instance, recent feeding or drinking can interfere with accurate measurement [3]. Additionally, using matrices related to short- or medium-term cortisol response to assess stress over an extended period would require repeated sampling [1,5], which can be challenging and may introduce confounding effects related to specific events and/or environmental disturbances [3,5,7], rather than giving a picture of the whole period. Due to the limitations of traditional methods in assessing chronic stress, hair cortisol concentration (HCC) has emerged as a valuable, non-invasive biomarker for monitoring long-term cortisol levels in both humans and animals [1,4,8]. In addition, HCC is known to remain stable for almost a year [9], particularly when stored in a dark environment [10], which highlights its practical advantage, and it is increasingly suggested as a valuable tool in clinical practice. Moreover, hair cortisol has been validated as a biomarker for chronic stress in dogs, with correlation with faecal and salivary cortisol levels [4,6,11]. Although the degree to which hair cortisol is derived from the circulation or from local synthesis in the hair follicle and whether it comes from the free fraction in plasma is still uncertain [5], it has been demonstrated that significant and prolonged stress may lead to a marked increase in HCC [12]. Moreover, a slower hair growth rate could result in prolonged cortisol incorporation into the hair [13]. However, to ensure its reliable application, it is essential to consider multiple possible influencing factors reported in literature with controversial results, including age, sex, hair colour and growth, body region, health status, diseases and environmental and management conditions [1,2]. In addition, cortisol levels may reflect both positive and negative arousal, making their possible interpretation as stress indicators more complex [14]. In some recent studies, HCC in dogs has also been examined in the context of the dog-guardian relationship. Notably, significant interspecies correlations in long-term stress have been observed, with human HCC strongly correlating with dog HCC [15]. Additionally, dog’s HCC was found to be primarily influenced by the guardian-dog relationship, although it was also associated with the guardian’s personality [16].

Given the growing use of HCC in stress and welfare research, we aimed to establish baseline HCC levels in healthy dogs (HD) and to investigate a range of factors that may influence it, including the presence of chronic disease conditions. To this end, we analysed HCC values with several variables available in our dataset, encompassing both dog- and guardian-related characteristics. Additionally, we analysed the association between HCC and HD’s guardian responses to a questionnaire assessing various aspects of their dogs’ state of welfare.

## 2. Materials and Methods

### 2.1. Participants

The study involved two groups of dog-guardian pairs: 128 Healthy Dogs (HD) and 25 dogs with a primary Chronic Gastroenteric Disease (CGD), along with their guardians.

#### 2.1.1. Healthy Dogs (HD) Group

At the time of selection for this study, all dogs underwent a veterinary clinical examination; only those assessed as healthy were included in the HD group. All subjects were recruited from the Veterinary Clinic “Dr. A. Bartoli” in Empoli (Italy). The primary reasons for which the dogs came to the clinic were annual vaccinations (93.8%) or pre-operative hematobiochemical analyses in preparation for neutering procedures (6.2%). As previously mentioned, anamnesis data were collected during the veterinary clinical examination; no current or past pathologies or ongoing treatments were identified. Hematobiochemical analyses and an abdominal ultrasound were conducted, but neither revealed any abnormalities. All the diagnostic assessments were performed after hair samples were collected.

The HD group consisted of a variety of breeds, as indicated in Table 1, with characteristics such as age, sex, neutering status and guardian information detailed in Table 2 and Table 3. The group of HD guardians included 78 women (60.9%) and 50 men (39.1%), with ages ranging from 19 to 80 years (mean ± standard deviation: 50.2 ± 14.3).

#### 2.1.2. Chronic Gastroenteric Disease (CGD) Dogs Group

All subjects were recruited at the time they were brought in for consultation at the Internal Medicine Service of the Veterinary Teaching Hospital at the University of Pisa and diagnosed with primary chronic gastroenteric disease, with clinical signs lasting for more than 3 weeks. Secondary forms (e.g., hypoadrenocorticism, pancreatic or hepatic diseases) were excluded, and cases were characterized using a stepwise diagnostic approach, as described in the literature [17]. A complete diagnostic workup (cell blood count, biochemical profile, urinalysis, and diagnostic imaging) was performed in these dogs to exclude other diseases. To further investigate, the medical records of all CGD patients at the time of hair sampling were reviewed to assess adrenal gland status based on abdominal ultrasound findings, which were performed by a veterinarian with expertise in diagnostic imaging.

In the CGD group, the dogs’ ages ranged from 1 to 14 years (mean ± standard deviation: 7.6 ± 3.9), comprising 13 females (52.0%, 8 entire and 5 neutered) and 12 males (48.0%, 8 entire and 4 neutered). The group of CGD guardians included 19 women (76.0%) and 6 men (24.0%), with ages ranging from 22 to 73 years (mean ± standard deviation: 44.5 ± 15.8).

Due to the lack of additional information from the questionnaire, in this study, the CGD group was only used for the comparison of HCC values.

### 2.2. Questionnaire

Guardians of the HD group dogs were asked to complete a questionnaire collecting the guardian’s (gender, age, occupation, and educational level) and dog information (name, age, breed, sex, and neutering status). Additionally, guardians were asked to assess their dog’s quality of life, stress level, and anxiety level on a 1–10 Likert scale [18,19]. They were also asked to evaluate how energetic/lively, happy/satisfied, active/serene, and calm/relaxed they perceived their dog to be over the past month on a 0–6 scale [20]. The questionnaire was provided to the caregiver in Italian and paper format to be completed at the time of the visit. All caregivers were adults (more than 18 yo) and signed the informed consent outlined in the questionnaire. The administered Italian questionnaire is available in the Supplementary Materials (S1).

### 2.3. Hair Samples Collection and Storage

These samples were obtained by shaving an area approximately 12 × 8 cm^2^ from the abdominal region with a veterinary electric clipper, during the preparation for ultrasound examination. The hair samples were stored on a paper sheet, sealed, and stored in a dry, dark environment for a maximum of 3 months.

### 2.4. Extraction of Cortisol from Hair Samples

To measure cortisol concentrations in hair samples, the protocol indicated in Mariti et al. [21], was followed with a slight modification in the washing procedure, consisting of using isopropanol instead of methanol. The main steps of the protocol:

1. Perform a coarse cleaning of the hair using tweezers on a white sheet of paper to remove all impurities and foreign materials; if the hair is long, cut the part closest to the root and use that for the analyses. Weigh 150 mg of hair and perform 2 washes with 3 mL of isopropanol each, followed by drying overnight under a fume hood.
2. Cut the hair sample with scissors for 1–2 min, and then, after including three zirconium beads for each sample, use the homogenizer performing 6 cycles at 4350 rpm for 30 s each. Weigh 50 mg of hair and proceed with the extraction (if extraction is not performed immediately, the samples can be stored in a dark environment).
3. Add 1 mL of methanol, vortex for 20 s, and then place on a shaker for 24 h (200 rpm).
4. After 24 h of shaking, centrifuge at 9000 rpm for 15 min and collect 0.6 mL of supernatant. Evaporate to dryness using nitrogen; if not proceeding immediately with the cortisol ELISA kit, freeze the samples.
5. Before beginning with the analysis, allow the kit and the samples to reach room temperature by leaving them out of refrigeration for approximately 90 min. Each hair sample was reconstituted with 200 µL of buffer [21]. Cortisol was measured using the Salimetrics^©^ (Carlsbad, CA, USA) enzyme immunoassay kit for high-sensitivity salivary cortisol. If cortisol levels exceed 3.0 μg/dl (82.77 nmol/L), the samples must be diluted with Assay Diluent and re-read to ensure accuracy; the final value is calculated by multiplying the measured result by the dilution factor. All samples were measured in duplicates, and the mean value was used for statistical analysis.

The validation parameters are based on a procedure described in a previous study and performed in the same laboratory [21].

### 2.5. Statistical Analysis

Despite the initial involvement of 130 HD subjects in the study, the statistical analyses included 128 samples, as two were excluded due to absorbance levels exceeding the standard range. Regarding the CGD group, the initial number of dogs was 29; however, four dogs were excluded because they were being treated with corticosteroids at the time of sampling or had been diagnosed with Addison’s disease (N = 25).

The data were analyzed by first assessing the normality of continuous variables using the Shapiro-Wilk test, in both HD and CGD group. Normal distribution assessment with the Shapiro-Wilk test revealed that HCC values (W = 0.85908, *p* = 1.09 × 10^−9^) and the dog’s age (W = 0.93845, *p*-value = 1.88 × 10^−5^) are not normally distributed. In HD, as data were not normally distributed, a non-parametric Mann–Whitney U test (*p* < 0.05) was performed to examine potential differences in HCC based on the guardian’s gender (male versus female) and on the dog’s neuter status (neutered vs. entire, regardless of sex); a non-parametric Kruskal–Wallis test (*p* < 0.05) with Bonferroni post-hoc correction was conducted to assess potential differences in HCC based on the dog’s sex (four groups: entire female, neutered female, entire male, neutered male), the guardian’s educational level (three groups: Elementary/Middle School, High School Diploma, Bachelor’s or higher Degree), the guardian’s occupation (seven groups: Employed, Self-employed professionals, Retired, Manual labourers, Worked directly with animals, Students and Unspecified jobs). A Spearman’s correlation test (*p* < 0.05) was conducted to assess the correlation between the HCC and both the dog’s age and the guardian’s age. A Multiple Linear Regression analysis (*p* < 0.05) was performed, creating a model with the dependent variable being the logarithm of HCC, and the independent variables including the dog’s age and sex (including neutered status), the guardian’s age, occupation, and educational level. After performing the Multiple Linear Regression, a backward stepwise selection was applied to refine the model. Additionally, an interaction between the guardian’s age and their occupation and educational levels was added to the model. To further investigate, a Spearman’s correlation test (*p* < 0.05) was also conducted to assess the correlation between the dog’s and the guardian’s age. An Ordinary Logistic Regression (ORL) analysis (*p* < 0.05) was performed with the dependent variables being Quality of life, Stress level, Anxiety level, Energetic/lively, Happy/satisfied, Active/serene, and Calm/relaxed scores, and independent variables being HCC. A Wilcoxon rank-sum test (*p* < 0.05) with continuity correction was used to compare cortisol levels, dog’s and guardian’s age in HD and CGD. The clinical reference range of hair cortisol concentration for all healthy dogs was calculated as 2.5th–97.5th percentiles while are used as the upper and lower limits of the reference interval (including the middle 95% of data) [22,23]. Descriptive statistics were performed, and the data are presented as median (interquartile range IQR and minimum-maximum values) unless otherwise noted.

## 3. Results

For the total included HD samples (N = 128), the HCC median was 6.41 pg/mg (IQR 4.66–8.69; min = 0.68, max = 28.98 pg/mg), whereas for CGD samples (N = 25), the HCC median was 4.79 pg/mg (IQR 3.33–5.51; min = 0.79, max = 9.09 pg/mg). The clinical reference range of HCC (2.5th–97.5th percentiles) was 1.62–14.96 pg/mg.

The descriptive statistics for the HD guardian’s age, dog’s age and canine HCC values are presented in Table 2.

The descriptive statistics for dog’s sex, guardian’s information and canine HCC values are presented in Table 3.

**Figure 1:**
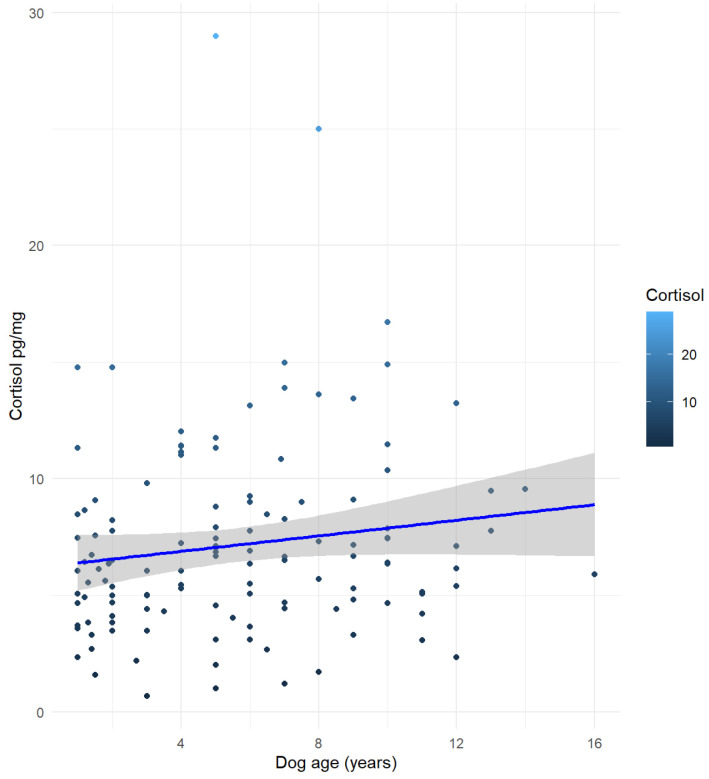
The Spearman’s correlation between Hair Cortisol Concentration (HCC) and the dog age (S = 287,258, *p* = 0.0442, rho = 0.1781).

**Figure 2:**
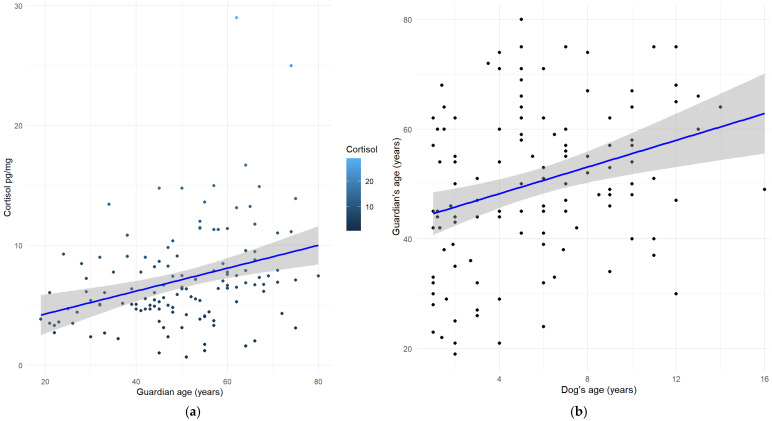
(**a**) The Spearman’s correlation between Hair Cortisol Concentration (HCC) and the guardian’s age (S = 229,603, *p* < 0.0001, rho = 0.3431); (**b**) Spearman’s correlation test between the dog’s age and the guardian’s age (S = 241,520, *p* = 0.0004, rho = 0.3089).

The non-parametric tests assessing the relationship between HCC and the dog’s sex (Kruskal-Wallis test, χ^2^ (3) = 1.6702, *p* = 0.6436), as well as the guardian’s gender (Mann-Whitney U test, W = 1828, *p*-value = 0.5545, r = −0.4091, 95% C.I. [−1.5533337; 0.7325464]), the dog’s neuter status (neutered vs. entire, regardless of sex; Mann-Whitney U test, W = 1965, *p*-value = 0.8266, r = 0.1463, 95% C.I. [−1.071051; 1.370745]), education level (Kruskal-Wallis test, χ^2^ (2) = 2.9521, *p* = 0.2285) did not reveal statistically significant results (*p* > 0.05). A Kruskal–Wallis test showed a significant difference in HCC among guardian’s occupation categories (χ^2^ (6) = 14.397, *p* = 0.0255); however, no pairwise comparisons reached statistical significance after applying the Bonferroni post-hoc correction. The Spearman’s correlation between HCC and the dog’s age (S = 287,258, *p* = 0.0442, rho = 0.1781) (Figure 1) and the guardian’s age (S = 229,603, *p* < 0.0001, rho = 0.3431) showed statistically significant results (Figure 2a).

Table 4 presents the significant results of the Multiple Linear Regression model examining the relationship between the logarithm dependent variable (HCC) and the independent variables (dog’s age and sex with neutered status, guardian’s age, occupation, and education level).

The model showed a residual standard error (SE) of 0.56 with 122 degrees of freedom. The multiple R^2^ was 0.1325, while the adjusted R^2^ was 0.09692. The overall model was statistically significant (F (5122) = 3.726, *p* = 0.004). The backward stepwise selection for the Multiple Linear Regression confirmed the significance of the model, as shown in Table 5.

The model had a residual standard error (SE) of 0.558 with 124 degrees of freedom. The multiple R^2^ was 0.1246, and the adjusted R^2^ was 0.1034. The model was statistically significant (F (3124) = 5.881, *p* = 0.0009). The Akaike Information Criterion (AIC) was −145.4.

Considering the significant results observed between HCC and the guardian’s age, an interaction between the guardian’s age and their occupation and educational levels was added to the model, but no significant results were obtained (interaction guardian’s age-occupation: Estimate = −0.0026, SE = 0.0018, *t*-value = −1.485, *p* = 0.140).

Additionally, Spearman’s correlation test showed a moderately positive correlation between the dog’s age and the guardian’s age (S = 241,520, *p* = 0.0004, rho = 0.31) (Figure 2b).

The following results were obtained with the analysis between HCC and the guardian-assessed evaluation scores, as shown in Table 6.

**Figure 3:**
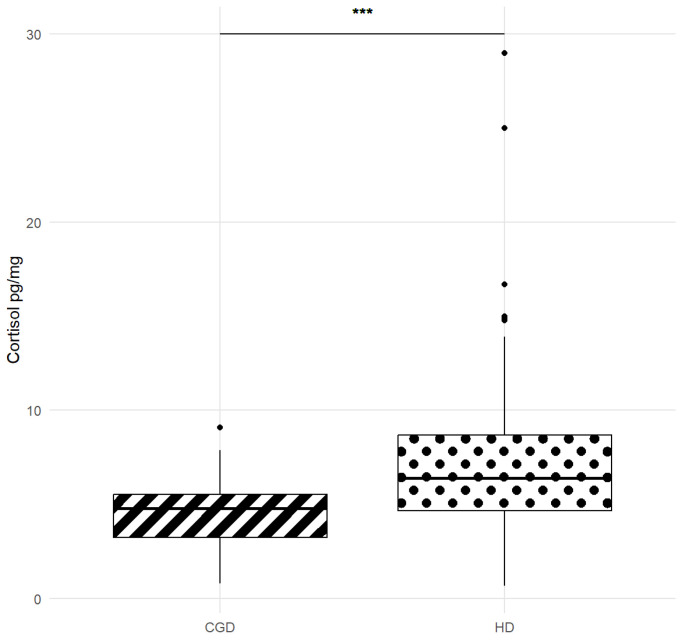
The Wilcoxon rank-sum test (W = 911, *p* < 0.001) with continuity correction between Hair Cortisol Concentration (HCC) in the chronic gastroenteric dogs group (CGD) and healthy dogs group (HD).

A significantly higher age was observed in chronically ill dogs compared to healthy dogs (Wilcoxon rank-sum test, W = 2061.5, *p* = 0.023, r = 2.0001, 95% C.I. [4.859126 × 10^−6^; 4.000013]). In contrast, no statistically significant difference was found in the guardians’ age between HD and CGD (Wilcoxon rank-sum test, W = 1134.5, *p* = 0.147, r = −5.999978, 95% C.I. [−13.000045; 1.999998]). A significantly lower cortisol concentration (Wilcoxon rank-sum test, W = 911, *p* = 0.0007, r = −2.0393, 95% C.I. [−3.2654845; −0.8784441]) was observed in chronically ill dogs (CGD and HD medians: 4.79 versus 6.41 pg cortisol/mg hair (Figure 3), despite their significantly higher age.

Additionally, among the 25 CGD dogs, only two showed ultrasonographic alterations of the adrenal glands: in one dog, they appeared reduced in size, and in the other, an altered adrenal echostructure due to the presence of a lesion was found.

## 4. Discussion

This study aimed to deepen the knowledge about hair cortisol concentration (HCC) in dogs, by evaluating the influence of some factors related to both dogs and guardians; some of the findings obtained in this study appear to be relevant to this objective.

Although hair cortisol concentration (HCC) is known to be unaffected by stressful events related to the sampling procedure and other short-term stressors or circadian rhythms [4,6], other influencing factors still exist. As indicated by Heimbürge et al. [1] and Mesarcova et al. [2], previous studies—often based on relatively small sample sizes—have provided controversial findings; some reported no significant differences in HCC regarding variables such as age [24], breed [6], sex or neuter status [6,25], whereas others suggested that these or additional factors—such as hair color [6,25], seasonality [15], nutritional status [25], well-being, and overall health [26,27]—could still influence HCC.

Sex-related differences in HCC appear to be inconsistent and may depend on behavioural, physiological, and metabolic factors [6,28], as several studies on dogs have reported no significant sex differences in HCC [6,24,26,27]. Other studies [15,16,29] conducted on smaller samples of dogs (with 58, 42 and 52 subjects, respectively) have reported higher HCC in females [12,13,23], whereas in the study by Bowland et al. [25], which included a much larger canine sample (454 subjects), the difference in HCC between males and females was considered inconclusive and attributed to the sexual dimorphism of individuals. In this study, HCC did not appear to be influenced by sex nor by neuter status.

Interesting results of this study concern the association of HCC with the age, both of the dog and of the guardian: a poor positive correlation was found between dogs’ HCC and their age; and a fairly [30] positive correlation between dogs’ HCC and their guardians’ age. This suggests that the dog’s HCC tends to increase with either the age of the dog and the age of the guardian, independently. Considering first the dog’s age as a factor influencing HCC, as reported by Mesarcova et al. in [2], previous studies have suggested that cortisol levels in the dog’s coat may increase with age. Goy-Thollot et al. [31] found that age had a highly significant effect on increasing plasma basal cortisol levels. In addition, Palazzolo and Quadri [32] reported that older dogs had a disrupted circadian rhythm of plasma cortisol, with a trend for higher mean cortisol levels observed in the old dogs group (mean age: 12.1 yo) compared to adults (mean age: 3.3 yo). However, this finding has not been confirmed by more recent research [6,28,33]. Additionally, although research on baboons has shown that HCC may increase again in advanced age [34], as stated by Heimbürge et al. in [1] the precise pattern of HCC changes appears to be species-specific and may involve a secondary rise in older individuals, likely due to alteration of the HPA axis. Findings from previous studies on the correlation of age on HCC appear inconsistent, with some reporting an age-related increase in cortisol levels, while others found no significant association. Although a clear explanation for the correlation between age and HCC could not be provided, the large sample size in the present study makes the observed result of particular interest. It suggests that the age of dogs (taken together with other factors, e.g., the age of the guardian) might be influential on HCC variation in dogs.

To further investigate these results, it may be useful to consider the moderately positive correlation found also between the dog’s and the guardian’s age, which indicated that older guardians tended to have older dogs. We hypothesized that dogs of older guardians likely engage in less physical activity, which is one of the other factors that may affect canine HPA axis activity and cortisol levels [1,2]. Although previous studies reported higher HCC in competition dogs than in companion or working dogs [26,28], they referred to highly active dogs, being involved in disciplines such as high level of protection work and flyball. Looking at basal levels of activity, in a study on humans higher volumes of daily sedentary behaviour among participants were associated with higher salivary cortisol levels [35]; although we did not collect data on the guardians’ levels of sedentary behaviour, it is likely that older adult persons are more sedentary than younger guardians. It would be interesting to investigate whether the association between low activity and higher cortisol levels could extend to increased sedentary behaviour in their dogs, potentially leading to higher HCC levels in these animals.

Other factors, which have been addressed in previous studies on dog’s HCC, include the body region, coat colour, and the guardian’s occupation. In our study, we consistently used the same body region for all dogs. As for the coat colour, not taken into consideration in the current analysis, literature reports conflicting findings: in fact, Bennett et al. [6] found differences in coat colour among a sample of German Shepherds and Labradors, while Nicholson et al. [24] reported no significant differences in a group that contained a greater variety of breeds, including crossbreeds, as in the present study. As for the guardian’s occupation, no significant differences were found across the different occupation categories, a result similarly reported by Sundman et al. [15]. However, given the correlation reported by Nicholson et al. [24] between dog’s HCC and the amount of time the dog spends alone at home, as well as the synchronization of HCC between dogs and their guardians described by Sundman et al. [15], it may be important to further investigate specific guardian-related aspects that influence the dog’s lifestyle.

Contrasting findings have been reported across other studies for the presence of chronic diseases. In the current study, the presence of a chronic disease influenced HCC, with lower HCC values observed in dogs from the CGD group compared to the healthy group. This finding is consistent with previous observations in dogs affected by epilepsy and anxiety, where HCC was higher in healthy individuals [26], likely due to a prolonged negative feedback mechanism of elevated cortisol levels and dysregulation of the HPA axis in chronically stressed subjects [26,36]. It can also be hypothesized that malabsorption and the gastrointestinal chronic inflammatory condition may influence the HPA axis [37,38]; however, additional studies are required to confirm this possibility.

In [26], dogs exhibiting greater signs of fear/anxiety showed lower hair cortisol concentration (HCC); similarly, in the present study, lower HCC was associated with an increased perception of anxiety in the dog by the guardian, suggesting a potential trend despite the lack of statistical significance, probably due to exhaustion and dysregulation of the HPA axis [26,36].

To further investigate these aspects, medical records were later reviewed, revealing ultrasonographic adrenal alterations in only two of the 25 CGD dogs; however, this result was not unexpected, as anatomical abnormalities of the adrenal gland cannot be unequivocally associated with functional alterations of the HPA axis [39]. Moreover, abdominal ultrasonography is commonly included in the diagnostic work-up of dogs with gastroenteric disorders, and it typically requires shaving the abdominal area. Hair from this area can be easily utilised for hair cortisol concentration (HCC) analysis, making the procedure both practical and minimally invasive [40]. A larger sample size is probably needed to obtain more robust and generalisable conclusions in chronically ill dogs. However, it must be noted that in the study by Nicholson et al. [24] involving 33 dogs, no significant difference in hair cortisol concentration was observed between chronically ill and healthy subjects, although the chronically ill dogs were significantly older, thus possibly being a confounding factor and suggesting the importance of involving sufficiently large samples to discern the impact of each single factor. Conversely, Park et al. [27] found that 26 dogs with atopic dermatitis had significantly increased hair cortisol levels compared to healthy controls. Similarly, in the human population, individuals with chronic pain were found to have higher hair cortisol concentrations than those not experiencing pain [41]. These findings suggest that both the type and severity of the chronic disease may affect HCC responses and should be taken into account in future assessments.

The main limitations of this study include the absence of data regarding coat colour, the dog’s lifestyle and care, and the length of time regularly left alone, all of which have been recognised as factors that can influence cortisol levels in previous research [6,24,26,28].

To the best of our knowledge, no previous studies have investigated the correlation between guardian’s age and canine HCC levels in such a large sample. Given recent findings highlighting the impact of the dog–guardian relationship on long-term stress (e.g., Höglin et al. [16]), including characteristics of the guardians as well as of the dog-guardian relationship may allow to further dive into the knowledge of factors influencing hair cortisol levels. Notably, previous studies measuring HCC in both dogs and their guardians, similarly to what has been observed for other variables, have provided controversial findings—e.g., Höglin et al. [16] found a long-term stress synchronisation between owners and their ancient/solitary hunting dog breeds, whereas Wojtaś et al. [42] did not find significant correlation between the hair cortisol levels of owners and their dogs—reflecting the complexity and variability in this field. Collecting HCC in both dogs and their guardians might also be helpful in findings not studied associations. Furthermore, the results from the comparison between healthy and chronically ill dogs emphasize the relevance of exploring these factors, with the aim of supporting the integration of such analyses into clinical practice.

## 5. Conclusions

The results of this study support the idea that hair cortisol concentration is shaped by a complex interplay of factors, some of which are already known and some need more exploration, using large samples and a multiparametric analysis. Using the latter approach, in this study the older age of the dog and the presence of a gastrointestinal chronic illness were significant factors, more than others, suggesting the relevance of considering more factors at the same time in order to avoid confounding effects possibly misleading the results. It is noteworthy that characteristics of the guardian, such as older age, might also be influential, and this suggests that future studies should focus on the collection of additional information concerning the dog, the guardian, and their relationship.

Overall, these findings suggest that, although HCC is an effective method for assessing cortisol levels over an extended period, its interpretation should be approached with caution due to the multiple variables that can impact it.

## Figures and Tables

**Table 1:** List of the breeds represented in the sample of N = 128 healthy dogs (HD). Each breed is represented the number of times indicated in the “Number of Dogs for Each Breed” column, together with the mean (±SD) for breeds with five or more subjects.

Breed	Number of Dogs for Each Breed	Mean (±SD)
Mixed-Breed	51	6.34 (±3.51)
Dachshund	7	10.39 (±8.52)
Labrador Retriever Jack Russell Terrier Cocker Spaniel	6	6.03 (±2.89) 8.46 (±2.04) 4.96 (±2.96)
Golden Retriever Poodle	5	5.08 (±0.46) 8.72 (±4.78)
Australian Shepherd English Setter	4	
Épagneul Breton Maremmano Sheepdog German Shepherd Beagle	3	
Italian Spinone Bolognese Dog Basset Hound Belgian Shepherd Lagotto Romagnolo	2	
Great Dane Cavalier King Charles Spaniel German Wirehaired Pointer Dogo Argentino German Hunting Terrier Miniature Pinscher Shiba Inu Boxer Siberian Husky Italian Volpino Springer Spaniel Border Collie	1	

**Table 2:** Descriptive statistics for the healthy dogs (HD) group: mean, standard deviation (SD), median, 25th–75th percentile range (IQR1, IQR3), variance (VAR), minimum, maximum, range, and Coefficient of Variation (CV).

HD Group	Guardian’s Age (Years)	Dog’s Age (Years)	Canine HCC (pg/mg)
Mean	50.2	5.6	7.16
Standard Deviation (SD)	14.3	3.6	4.18
Median	50.0	5.0	6.41
IQR1	41.8	2.0	4.66
IQR3	60.5	8.1	8.69
VAR	204.2	13.0	17.44
Min	19.0	1.0	0.68
Max	80.0	16.0	28.98
Range	61.0	15.0	28.31
Coefficient of Variation (CV)	0.3	0.6	0.58

**Table 3:** Descriptive statistics for the healthy dogs (HD) group: dog’s sex and guardian’s information (gender, educational level and occupation), including sample size (N), percentage of the total sample (%), median with 25th–75th percentile range (IQR1; IQR3), and minimum and maximum HCC values.

**Dogs (N = 128)**			**Canine HCC (pg/mg)**		
	**N**	**%**	**Median (IQR1; IQR3)**	**Min**	**Max**
Sex					
Female (entire + neutered) Female entire Female neutered	67	52.3	7.10 (4.67; 9.09)	0.68	28.98
	29	22.7	6.04 (4.66; 9.55)	2.19	28.98
	38	29.7	7.10 (4.68; 9.06)	0.68	25.00
Male (entire + neutered) Male entire Male neutered	61	47.7	6.35 (4.55; 7.77)	1.02	14.98
	51	39.8	6.42 (5.03; 7.99)	1.02	14.98
	10	7.8	5.26 (4.13; 6.48)	1.72	11.48
Neutered (male + female) Entire (male + female)	48 80	37.5 62.5	6.42 (4.61; 9.02) 6.41 (4.68; 8.31)	0.68 1.02	25.00 28.98
**Guardian (N = 128)**			**Canine HCC (pg/mg)**		
	**N**	**%**	**Median (IQR1; IQR3)**	**Min**	**Max**
Gender					
Female Male	78	60.9	6.46 (4.58; 7.68)	1.60	25.00
	50	39.1	6.38(4.67; 9.06)	0.68	28.98
Education level					
Elementary or Middle School High School Diploma Bachelor’s or higher Degree	20	15.6	5.58 (3.83; 7.28)	1.21	28.98
	68	53.1	6.35 (4.68; 9.02)	0.68	25.00
	40	31.3	7.13 (4.94; 9.28)	2.19	16.70
Occupation					
Employed Self-employed professionals Retired Manual labourers Worked directly with animals Students Other	30	23.4%	7.81 (5.08; 9.10)	2.36	14.76
	23	18.0%	6.67 (4.54; 8.21)	1.72	25.00
	21	16.4%	7.10 (6.14; 11.02)	1.60	16.70
	19	14.8%	6.42 (3.99; 7.84)	0.68	14.76
	8	6.3%	4.61 (3.48; 5.39)	1.02	10.36
	5	3.9%	3.59 (3.31; 3.83)	2.69	6.04
	22	17.2%	5.90 (4.67; 7.67)	2.19	28.98

**Table 4:** Summary of the Multiple Linear Regression Model. The table reports the estimated coefficients (Estimate), standard errors (SE), *t*-values, and corresponding *p*-values (Pr (>|t|)) for each predictor Variable included in the model, assessing their association with the dependent variable, log-transformed hair cortisol concentration (log HCC). Significance codes: ‘***’ 0.001; ‘**’ 0.01.

Variable	Estimate (β)	SE (Standard Error)	*t*-Value	Pr (>|t|)
Intercept	1.619530	0.265339	6.104	1.27 × 10^−8^ ***
Dog’s age	0.010134	0.015061	0.673	0.50228
Dog’s sex	−0.053461	0.057238	−0.934	0.35214
Guardian’s age	0.011936	0.003724	3.205	0.00172 **
Occupation	−0.033424	0.024930	−1.341	0.18250
Education	−0.119572	0.079009	−1.513	0.13276

**Table 5:** Summary of the Multiple Linear Regression model obtained after backward stepwise selection. The table reports the estimated coefficients (Estimate), standard errors (SE), *t*-values, and corresponding *p*-values (Pr (>|t|)) for predictor variables included in the final model, assessing their association with the dependent variable, log-transformed hair cortisol concentration (log HCC). Significance codes: ‘***’ 0.001.

Variable	Estimate (β)	SE (Standard Error)	*t*-Value	Pr (>|t|)
Intercept	1.514083	0.220353	6.871	2.74 × 10^−10^ ***
Guardian’s age	0.012826	0.003492	3.672	0.000356 ***
Occupation	−0.038260	0.024364	−1.570	0.118888
Education	−0.113831	0.076526	−1.487	0.139426

**Table 6:** Ordinal Logistic Regression (OLR) coefficients for cortisol as a predictor of guardian-assessed evaluation scores in healthy dogs. β = regression coefficient, indicates that for every 1-unit increase in Hair Cortisol Concentration (HCC), the odds of a dog moving to a higher score of the outcome increase by a certain amount, in terms of log-odds; SE = standard error; z = the ratio of the estimated coefficient (β) to its standard error (SE). Significance codes: ‘.’ 0.1.

Outcome	β (Cortisol)	SE	z	*p*-Value
Energetic/lively	0.0486	0.0442	1.099	0.272
Happy/satisfied	0.0362	0.0431	0.840	0.401
Active/serene	0.0302	0.0421	0.718	0.473
Calm/relaxed	−0.0350	0.0374	−0.936	0.349
Quality of life	0.02603	0.0414	0.629	0.529
Stress level	−0.03899	0.0393	−0.991	0.322
Anxiety level	−0.06730	0.0393	−1.711	0.087(.)

## Data Availability

Dataset is available upon request from the authors.

## Supplementary Materials

The following supporting information can be downloaded at: https://www.mdpi.com/article/10.3390/ani15131901/s1, Questionnaire S1: The administered Italian questionnaire.
