# Supplementary material for: Factors Affecting Hair Cortisol Concentration in Domestic Dogs: A Focus on Factors Related to Dogs and Their Guardians
Source: Animals (Basel). 2025 Jun 27;15(13):1901. doi: 10.3390/ani15131901 (PMC12248899; doi:10.3390/ani15131901)
Supplement: Supplementary file 1 [file animals-15-01901-s001.zip › animals-3685203-supplementary.pdf]

**Supplementary material S1**

**The administered Italian questionnaire**

If you have read the information notice and agree to participate in this project, please mark "YES":

□ YES

□ NO

Date ………………..

Alphanumeric Code ………………..………………..

**GUARDIAN'S INFORMATION**

Gender:

□ Female

□ Male

□ Other/Prefer not to specify

Guardian's age: ………………..

What is your job? (Multiple selectable answers)

□ Work with animals (veterinarian, dog trainer, dog breeder, etc.)

□ Employed

□ Self-employed professionals

□ Manual labourers

□ Students

□ Unspecified jobs

□ Other

Educational Qualification:

□ Bachelor's or higher Degree

□ High School Diploma

□ Elementary/Middle School

**DOG'S INFORMATION**

Name: ………………..………………..

Sex: ………………..……………………

Age: ………………..…………………..

Breed: ………………..…………………

The following questions refer to the quality of life of your dog and the impact he/she (or it) has had on the entire family over the past month.

Please, read the questions carefully and select one answer for each.

How would you rate the quality of life of your dog on average over the past month?

1 = poor, 10 = excellent   ……………

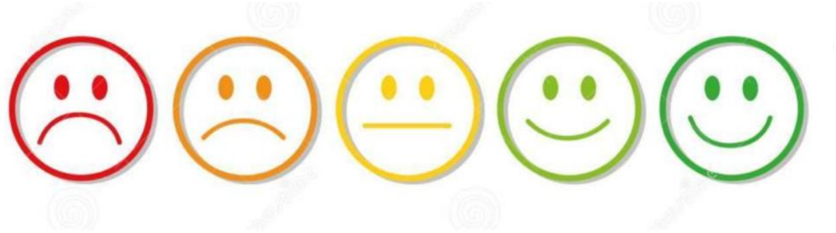

How would you rate your dog's stress level on average over the past month?

1 = not (at all) stressed, 10 = extremely stressed   ……………

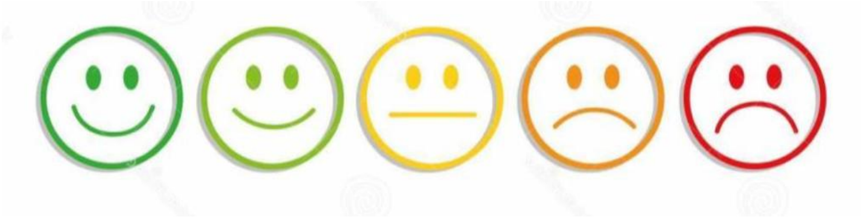

How would you rate your dog's anxiety level on average over the past month?

1 = not (at all) anxious, 10 = extremely anxious   ……………

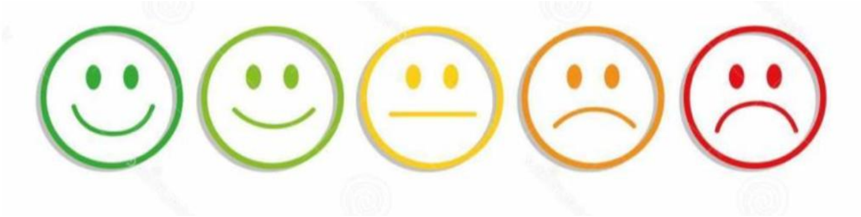

Over the past month, your dog has been (to 0 = not at all, at 6 = it couldn’t be more):

| Adjectives | Scores |
|---|---|
| Energetic/lively | |
| Happy/satisfied | |
| Active/serene | |
| Calm/relaxed | |
